# Human perinatal stem cell derived extracellular matrix enables rapid maturation of hiPSC-CM structural and functional phenotypes

**DOI:** 10.1038/s41598-020-76052-y

**Published:** 2020-11-04

**Authors:** Travis Block, Jeffery Creech, Andre Monteiro da Rocha, Milos Marinkovic, Daniela Ponce-Balbuena, Eric N. Jiménez-Vázquez, Sy Griffey, Todd J. Herron

**Affiliations:** 1 StemBioSys, Inc, 3463 Magic Drive, Suite 110, San Antonio, TX 78229 USA; 2 Frankel Cardiovascular Regeneration Core Laboratory, University of Michigan, North Campus Research Complex, 2800 Plymouth Road, B26 223N, Ann Arbor, MI 48109 USA; 3 Cartox, Inc, 1600 Plymouth Road, B520 2nd floor, Ann Arbor, MI 48109 USA; 4 Department of Comprehensive Dentistry, University of Texas Health at San Antonio, San Antonio, TX USA

**Keywords:** Biotechnology, Cell biology, Drug discovery, Pluripotent stem cells

## Abstract

The immature phenotype of human induced pluripotent stem cell derived cardiomyocytes (hiPSC-CMs) is a major limitation to the use of these valuable cells for pre-clinical toxicity testing and for disease modeling. Here we tested the hypothesis that human perinatal stem cell derived extracellular matrix (ECM) promotes hiPSC-CM maturation to a greater extent than mouse cell derived ECM. We refer to the human ECM as Matrix Plus (Matrix Plus) and compare effects to commercially available mouse ECM (Matrigel). hiPSC-CMs cultured on Matrix Plus mature functionally and structurally seven days after thaw from cryopreservation. Mature hiPSC-CMs showed rod-shaped morphology, highly organized sarcomeres, elevated cTnI expression and mitochondrial distribution and function like adult cardiomyocytes. Matrix Plus also promoted mature hiPSC-CM electrophysiological function and monolayers’ response to hERG ion channel specific blocker was Torsades de Pointes (TdP) reentrant arrhythmia activations in 100% of tested monolayers. Importantly, Matrix Plus enabled high throughput cardiotoxicity screening using mature human cardiomyocytes with validation utilizing reference compounds recommended for the evolving Comprehensive In Vitro Proarrhythmia Assay (CiPA) coordinated by the Health and Environmental Sciences Institute (HESI). Matrix Plus offers a solution to the commonly encountered problem of hiPSC-CM immaturity that has hindered implementation of these human based cell assays for pre-clinical drug discovery.

## Introduction

Human induced pluripotent stem cell-derived cardiomyocytes (hiPSC-CMs) can be produced in virtually unlimited quantities with high purity and offer a model system to “humanize” and hasten preclinical drug development^1–4^. Importantly hiPSC-CMs are commercially-available in cryopreserved vials for immediate use. In 2018, an international multisite validation study of hiPSC-CMs demonstrated their potential to detect drug-induced proarrhythmic effects as part of the Comprehensive In Vitro Proarrhythmia Assay (CiPA) paradigm^5^. In fact, the FDA has considered using hiPSC-CMs for a new approach called *“Clinical Trials in a Dish”,* based on the concept of screening for cardiac safety and toxicity at the level of a representative population^6,7^. Despite the validated utility of hiPSC-CM based cardiotoxicity assays, widespread adoption of this new paradigm has faced resistance because of the immature, fetal-like phenotypes of the cells available to date^8,9^. Maturation of hiPSC-CM phenotypes in high-throughput screening (HTS) assays is required to support revision of international pre-clinical cardiotoxicity screening guidelines to include this innovative human cell-based approach.

Current approaches to hiPSC-CM maturation include: long term culture of at least 30–100 days^10^, exercising cells mechanically and electrically^11,12^, metabolic and hormonal manipulations^13^, co-culture with other cell types^14^, 3D organoid development^15,16^ and modulation of extracellular matrix (ECM) properties^17^. Of these approaches ECM manipulation in 2D hiPSC-CM monolayers offers a highly feasible option with quantifiable variables that are compatible with high throughput screening assays. Here, we present Matrix Plus-a novel human in vitro perinatal stem cell-derived ECM that supports rapid (7 days) structural and functional maturation of hiPSC-CM phenotypes in 2D monolayer culture. In our study the effects of Matrix Plus ECM on hiPSC-CM phenotypes are compared to other commercially available ECM, including the commonly used Matrigel ECM. The utility of Matrix Plus coated 96 well plates for hiPSC-CM proarrhythmia assays is validated using clinically relevant doses of medications with known risk to cause arrhythmias as outlined in the recent hiPSC-CM validation assay.

## Materials and methods

### Preparation of human perinatal stem cell derived ECM

Human ECM coated plates were produced under aseptic conditions using procedures adapted from Chen et al.^18^ Commercially-available amniotic fluid was purchased from Life Line Stem Cell (New Haven, IN). Life Line Stem Cell procures amniotic fluid and other perinatal tissues with informed consent at various hospitals under IRB approval (Life Line Stem Cell Review Board). Briefly, perinatal stem cells isolated from perinatal tissue following planned c-section, were isolated on CELLvo Matrix (StemBioSys, San Antonio, TX) and cultured for two passages before cryopreservation. Cryopreserved cells were thawed and expanded for an additional two passages before seeding onto fibronectin-coated 6-well tissue culture plates and cultured to confluence in alpha-minimum essential medium (αMEM) modified without phenol red, and supplemented with anti-anti, 2 mM GlutaMAX, and 15% v/v fetal bovine serum (FBS). All methods with human samples were carried out in accordance with relevant guidelines and regulations of the National Institutes of Health. At confluence, full medium was replaced with new medium supplemented with 50 µM ascorbic acid to induce matrix secretion. After 3 days culture in the inducing medium, media was aspirated, plates were washed once with PBS, and then incubated, for at least 7 min, at room temperature, in PBS containing 0.5% (v/v) Triton-X-100 with 20 mM NH_4_OH (to decellularize ECM). The decellularized ECM was washed thoroughly with PBS and deionized water to remove the detergent before being allowed to air-dry in a biosafety cabinet. Once the intact decellularized ECM was dried, the plates were stored at 4 °C until use. Air-dried, ECM-coated plates have a shelf-life of at least 12 months when stored at 4 °C. Before use, plates were rehydrated with PBS at 37 °C for 1 h. Upon rehydration, ECMs swell into a thin hydrogel. From a single donor, we may isolate enough perinatal stem cells to produce more than 12,000 96-well plates. We refer to this new human cell derived ECM as Matrix Plus, and it is now commercially available (StemBioSys, Inc San Antonio, TX).

### Determination of structural and mechanical properties by atomic force microscopy

Physical and structural properties of the matrices were analyzed with a NanoScope Catalyst (Bruker) atomic force microscope (AFM) mounted on a Nikon Ti Inverted Epifluorescence Microscope in the PeakForce Quantitative Nanomechanical Mapping (PF-QNM) mode. The probes and matrices were immersed in PBS. At least 5 randomly-selected, 100 × 100 mm, fields were scanned for each group.

### Determination of matrix composition by mass spectrometry

ECM proteins were extracted according a modification of the approach outlined before^19^. Methods description, adapted from the original method description is provided here in detail to ensure reproducibility of results in other laboratories. ECM was initially dispersed by stirring and sonication, performed in PBS. The protein suspension was then centrifuged, and the resulting pellet treated with Protein Extraction Reagent Type 4 (Millipore-Sigma), sonicated 30 min (on ice) and then dried under vacuum. To reconstitute protein, Ready Prep 2-D Rehydration Sample Buffer (Bio-Rad) was added to the dried ECM pellet, and the suspension subjected to several sonication (on ice) and vortexing (32 °C) cycles. The ECM samples were then processed through two liquid nitrogen freezing/thawing cycles, followed by centrifugation for 10 min at 21,000 × g. The supernatant was removed from the resulting pellet.

The supernatant (Supernatant #1) was adjusted to 8 M urea concentration. The pellet was dissolved in 100 µL DMSO, sonicated for 30 min on ice, followed by re-centrifugation for 10 min at 21,000 × g. The supernatant yielded by this centrifugation step (Supernatant #2) was also adjusted to 8 M urea concentration. Supernatants #1 and #2 were then mixed and centrifuged to produce a new supernatant (#3). 1 mL Acetone was combined with a 200µL aliquot of Supernatant #3, vortexed to mix and stored at 4 °C overnight. The mixture was then centrifuged briefly to separate and remove the organic supernatant (acetone). The resulting pellet was dried at room temperature and then reconstituted in 50µL SDS buffer (2x), boiled at 100 °C for 5 min and loaded onto an SDS-PAGE gel.

Electrophoretic protein separation was performed using standard, one-dimensional, SDS-PAGE with Coomassie blue staining. Trypsin digestion (Promega) was performed in situ, to release protein from discrete serial sections of each lane. Digests were analyzed using capillary HPLC-electrospray ionization tandem mass spectrometry (HPLC–ESI–MS/MS) on a Thermo Fisher LTQ fitted with a New Objective PicoView 550 nanospray interface. On-line HPLC separation of the digests was accomplished with an Eksigent NanoLC micro HPLC. A mass spectral scan strategy was used in which a survey scan was acquired followed by data-dependent collision-induced dissociation (CID) spectra of the seven most intense ions in the survey scan. Mascot (Matrix Science) was used to probe the mass spectra against the SwissProt database. Methionine oxidation was considered as a variable modification. Cross correlation of the Mascot results with X! Tandem and determination of protein and peptide identity probabilities were accomplished by Scaffold (Proteome Software; https://www.proteomesoftware.com/products/scaffold/). Protein identifications were accepted using the following criteria: minimum number of peptides, 2; peptide probability, ≥ 95%; protein probability, ≥ 99%. Similar methods have been employed to describe protein composition of other types of ex vivo prepared matrices^20,21^.

It is important to note, that there is some degree of batch-to-batch variability that may be caused by donor differences in the cells used to produce the matrix or by random variability introduced by cell culture and the decellularization process. Principal component analysis based on biochemical composition reveals tight clustering in Matrix Plus composition, relative to CELLvo Matrix (supplemental figure 1). For this manuscript’s, only proteins identified in every batch of a Matrix Plus analyzed were considered relevant. In general, only proteins present in exceptionally low abundance were not expressed by all donors.

### Plating hiPSC-CMs and EP/cardiotoxicity assay

MatrixPlus coated 6 or 96 well plates were stored at 4 °C, transferred to room temperature on the day of cell plating and rehydrated (30 min) in Hank’s Balanced Salt Solution (HBSS). HBSS was aspirated and hiPSC-CMs were plated essentially as before^17,22^. Briefly, commercially available iCell^2^ Cardiomyocytes (Cellular Dynamics International) were thawed at 37 °C for 4 min, diluted in 7 ml of EB20 medium supplemented with blebbistatin (DMEM:F12, 20%FBS, 0.1% glutamine, 0.2%NEAA, 25 µM blebbistatin) and centrifuged (1000 rpm for 5 min) for removal of cryoprotectants. All work with human pluripotent stem cell derived cardiomyocytes was performed with approval from the University of Michigan Human Pluripotent Stem Cell Research Oversight committee (HPSCRO). All methods with human samples were handled following NIH guidelines. Cells from a single vial were diluted in EB20 with blebbistatin (1.106 cells/mL) and split evenly for plating on Matrigel, CELLvo Matrix and MatrixPLUS. Total amount of cells per functional syncytia varied depending on plate format, six well plates received 150,000 cells in the center of 1 cm diameter of matrix; and 96 well plates received 75,000 cells per well. Lower density plating (25,000 cells per well) was also used to more clearly observe cellular shape. hiPSC-CMs were maintained in cardiomyocyte EB20 supplemented with blebbistatin for 2 days before culture in RPMI supplemented with B27 for another 5 days^1^.

hiPSC-CM monolayer membrane potential was recorded from hiPSC-CMs plated on different matrices using the current-clamp mode of the MultiClamp 700B amplifier and the Digidata 1440A digitizer (Molecular Devices, Sunnyvale, CA), essentially as before^17,23^. Briefly, borosilicate glass pipettes of resistances ranging from 4 to 6 MΩ were filled with an intracellular pipette solution containing (in mmol/L): MgCl2 (1), EGTA (1), KCl (150), HEPES (5), phosphocreatine (5), K2ATP (4.46), β-hydroxybutyric acid (2), and adjusted to a pH of 7.2 with KOH. Functional syncytia were perfused with a warm (37 ± 1.5 °C) external solution adjusted to pH 7.4 with NaOH, which contained (in mmol/L): NaCl (148), NaH2PO4 (0.4), MgCl2 (1), glucose (5.5), KCl (5.4), CaCl2 (1.8) and HEPES (15). Formation of a GΩ seal was followed by rupture of the patch membrane for recording of spontaneous action potentials. Data acquisition was performed using pCLAMP software (version 10.3; Molecular Devices, Sunnyvale, CA; https://www.moleculardevices.com/applications/patch-clamp-electrophysiology). Action potential recordings were made in different regions of the monolayer (3–5 per monolayer).

### Proarrhythmia screen in 96 well plates using CiPA compounds

Proarrhythmia screening assay was performed using voltage sensitive dye (VSD, FluoVolt) and high speed CCD camera (200fps)^24^. Briefly, cells were loaded with VSD (1:1000 FluoVolt and 1:100 Powerload in HBSS) for 15 min inside of a cell culture incubator before being exposed to drugs listed as high, intermediate and low risk for arrhythmias for 30 min in the incubator^5^. Supplemental Tables 1–3 outline each drug tested, the CiPA risk classification, the concentrations used and the clinical recorded Cmax for each drug to provide clinical reference for drug concentrations. Cells were then submitted to optical mapping for detection of voltage changes recorded with the CCD camera via selection of appropriate light wavelength (filter 515 nm, green light, Chroma). Movies were analyzed with custom made analysis software (Scroll) for determination of action potential duration and conduction velocity as described before^22,24,25^.

### hiPSC-CM structural analysis

hiPSC-CM morphology and monolayer orientation was monitored by live cell phase contrast and fluorescence microscopy (Incucyte Zoom, Essen Bioscience, Ann Arbor, MI). Confocal microscopy and immunostaining for hiPSC-CM using commercially available antibodies, listed here. Briefly cells were fixed with 2% paraformaldehyde for 10 min and washed 3 times with PBS before permeabilization with PBS supplemented with 0.1% of Triton-X (permeabilization buffer). After permeabilization, non-specific biding was blocked with 10% of normal donkey serum in permeabilization buffer (blocking buffer) for 30 min. Blocking buffer was replaced with blocking buffer containing primary antibodies for 60 min at room temperature. Samples were washed 3 times with permeabilization buffer previously to incubation with secondary antibodies diluted in blocking buffer for 45 min at room temperature and protected from light. Following, secondary antibodies were removed, and samples were incubated with DAPI and DAPI/Phalloidin for 5 min in the dark and washed for 3 times with PBS. Stained samples were mounted on a coverslip and stored in the dark until confocal microscopy imaging. For mitochondrial distribution and functional assays, hiPSC-CM monolayers were stained with MitoTracker RedCMXRos (50 nM, 30 min in HBSS). Live cell imaging of mitochondria was done using a Nikon A1R laser scanning confocal microscope with green excitation and red emission. Murine ventricular cardiomyocytes were obtained with full institutional approval (UCUCA of the University of Michigan) and were stained with MitoTracker RedCMXRos for comparison to hiPSC-CMs. All animal handling was carried out in accordance with institutional and federal regulations and guidelines. The cell isolation procedure has been described recently in detail^26^. Briefly, mice (2–4 mo old) were injected with 1 mL heparin (100 IU/mL ip) 20 min before heart excision. Animals were anesthetized with a mixture of ketamine (116 mg/kg ip) and acepromazine (11 mg/kg ip). The heart was removed quickly from the chest and retrogradely perfused through the aorta at a constant flow (4 mL/min) at 37 °C for 4 min with a Ca^2+^-free buffer containing (in mmol/L) 113 NaCl, 4.7 KCl, 1.2 MgSO_4_, 0.6 Na_2_HPO_4_, 0.6 KH_2_PO_4_, 10 KHCO_3_, 12 NaHCO_3_, 10 HEPES, 10 2.3-butanedione monoxime (BDM, Sigma), 30 taurine, and 5.5 glucose. All solutions were filtered (0.2-µm filter) and equilibrated with 100% O_2_ for at least 20 min before use. Enzymatic digestion was initiated by adding collagenase type II (773.4 U/mL; Worthington), trypsin (0.14 mg/mL), and CaCl_2_ (12.5 µmol/L) to the perfusion solution. After 6–8 min of digestion, the ventricles were removed and single cells isolated by physical separation. Isolated murine myocytes were plated on laminin coated coverslips for 1 h, then stained for mitochondria and images were taken on the same day. Further comparisons on mitochondrial function were performed with live cell staining with JC-1 according to manufacturer’s recommendation and imaged using a laser scanning confocal microscope or high content live cell fluorescent microscope (Incucyte, Sartorius). Data is presented as the red/green fluorescence ratio.

### Western blotting

Samples were lysed in 2 × laemmli buffer prior loading of 15µL of lysate per well of a NUPAGE-SDS gel for electrophoresis for 2 h at 100 mA. Samples were transferred to a nitrocellulose membrane with a semi-dry apparatus for 3 h at 90 mA. Nitrocellulose membranes were blocked in PBS with 0.1% Tween 20 and 5% of skim milk (WB blocking buffer) for 1 h under agitation at room temperature. WB blocking buffer was replaced with WB blocking buffer containing primary antibodies and incubated at 4 °C overnight. Subsequently membranes were washed 3 times with PBS with 0.1% Tween 20 and incubated with the secondary antibody diluted in WB blocking buffer. Antibody information is included in supplemental Table 4. Membranes were prepared for development with 3 washes with PBS with 0.1% Tween 20 and addition of chemiluminescent reagent (ECL Pierce or West Pico) for image capture with a Chemidoc and image density analysis with Imagelab (BioRad). As a positive control for cTnI expression, human cardiac ventricular homogenate was loaded (supplemental Fig. 7) on the gel. Human cardiac protein homogenate was prepared from biopsy samples collected with informed consent and with University of Michigan IRB approval from unused transplant donor heart collected by Gift of Life Michigan.

## Results

### Human perinatal stem cell derived ECM characterization

**Figure 1:**
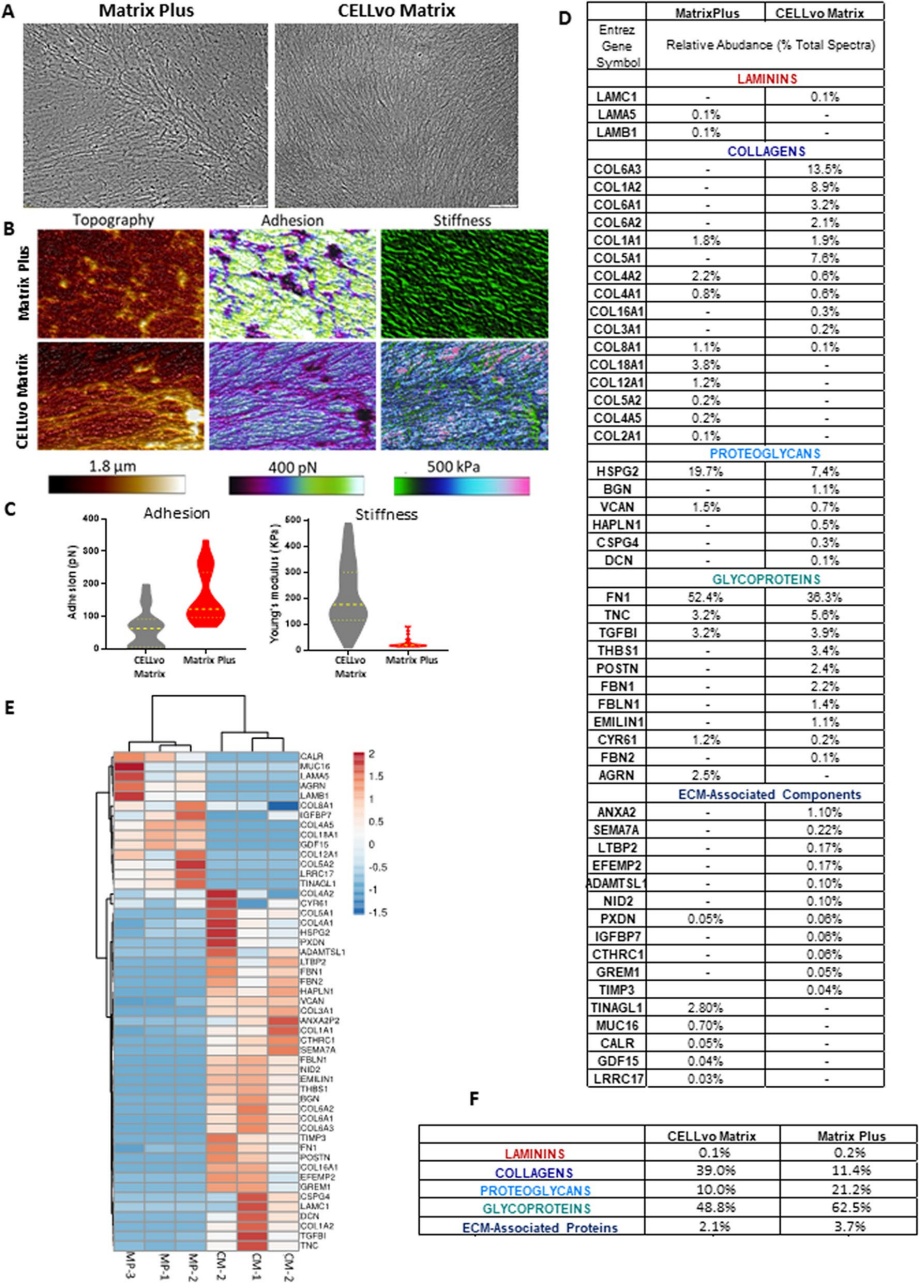
Physical and biochemical characterization of Matrix Plus and CELLvo Matrix. (**A**) Representative phase contrast images (100 × magnification) of Matrix Plus and CELLvo Matrix coated in plastic bottom multi-well dishes show that both matrices have a similar, naturally-aligned, fibrillar architecture. (**B**) Representative atomic force micrographs showing differences in topography, adhesion, and stiffness of Matrix Plus and CELLvo Matrix. Each image represents a 40 × 40um region of the matrix. (**C**) Adhesion and stiffness (elastic modulus) of Matrix Plus and CELLvo Matrix as measured by atomic force microscopy. Each data point represents one randomly selected independent measurement. For adhesion and stiffness, p < .001 or .00001, respectively. (**D**) Detailed composition of Matrix Plus and CELLvo Matrix reveal differences in the composition of the two matrices, as determine by mass spectrometry. Values reported are in terms of percent of total spectra after removing contribution of intracellular proteins and residual serum from the matrix production process. The values are averaged for n = 3 biologic replicates in each group. (**E**) Heat map of biochemical composition as determined by mass spectrometry. CM-1, CM-2, and CM-3 represent lots of CELLvo Matrix produced by unique donors. MP-1, MP-2, and MP-3 represent lots of Matrix Plus produced by unique donors. Rows are centered and unit variance scaling is applied to rows. Both rows and columns are clustered without bias using correlation distance and average linkage. This analysis shows similarities in composition across donors for each matrix type. (**F**) Composition of the two matrices is broken down by type of matrix component. CELLvo Matrix is more collagen-rich and less proteoglycan rich, relative to Matrix Plus.

Matrix Plus was generated from human perinatal stem cells using methods similar to those established with other matrix-producing cells^27^. Matrix Plus ECM characterization data is presented in Fig. 1. To provide context, Matrix Plus is characterized alongside another commercially available human ECM derived using human bone marrow cells called CELLvo Matrix. Each ECM is produced using the same process, only the starting cell type for ECM secretion is different. Importantly, the cardiomyocyte maturation phenomenon present on Matrix Plus is not observed on CELLvo Matrix, suggesting that any differences are potentially related to the mechanism of action driving the maturation process. In the very broadest sense, the two matrices (Matrix Plus & CELLvo Matrix) are both composed of the same types of proteins laid down in organized, semi-aligned, fibrillar structures. However, brightfield microscopy shows that, relative to Matrix Plus, fibrillar structures in CELLvo Matrix appear more prominent and tightly organized (Fig. 1A). Atomic force microscopy (AFM) showed that Matrix Plus has unique structural and mechanical characteristics including (Fig. 1B,C): lower roughness, adhesion and Young’s modulus compared to CELLvo Matrix.

The observed disparities in physical and mechanical properties among the matrices suggest differences in the underlying biomolecular composition. Biochemical analysis showed that Matrix Plus is enriched in several classes of ECM components (Fig. 1F) including increased proteoglycans but contains less total collagen (percent of total composition relative to CELLvo Matrix). While a molecular proteomic analysis of every compositional variation is beyond the scope of the current manuscript, Fig. 1D–F provides a more detailed catalogue of matrix components. As discussed elsewhere, the major collagens of CELLvo Matrix are collagens type VI and type I. Significantly, collagen VI was entirely absent in Matrix Plus and only small quantities of one subunit of collagen I were detected. This was a surprising finding, given the ubiquity of collagen I in stromal tissue and the well-documented importance of collagen VI for maintaining progenitor niches^28,29^. Another significant difference was the abundance of perlecan in Matrix Plus.

### Matrix plus ECM promotes hiPSC-CM functional maturation

**Figure 2:**
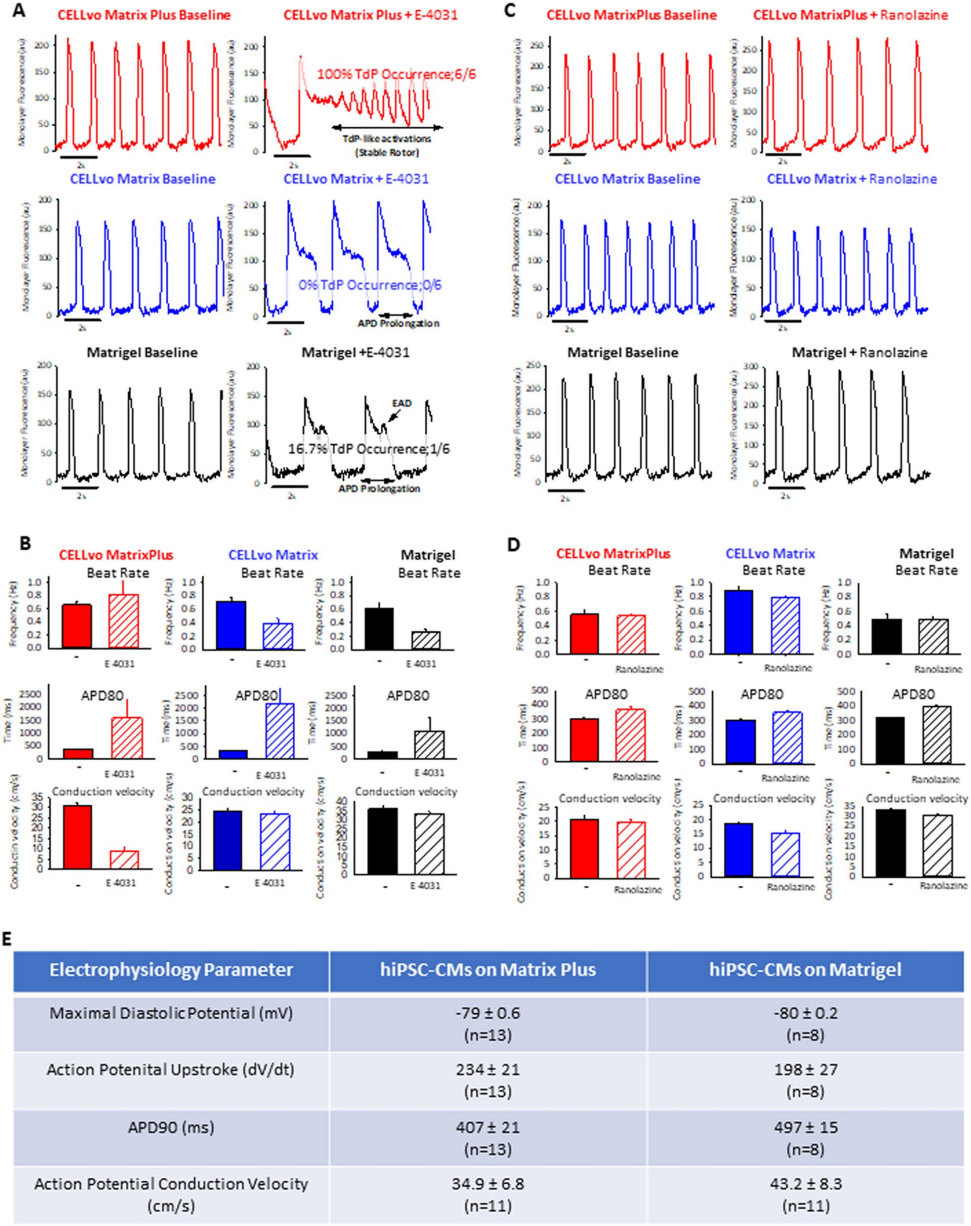
Effects of ECM on electrophysiology and drug-response of hiPSC-CMs. (**A**,**B**) hiPSC-CMs cultured on Matrigel, CELLvo or Matrix Plus were treated E-4031 (500 nM). Monolayers plated on Matrix Plus did not have a significant change in beat rate (n = 6, Δ beat rate =  + 0.22 Hz; p = 0.343), but had a significant increase in APD80 (n = 6, Δ APD80 =  + 1,227.5 ms; p = 0.002) formation of rotors (TdPs) in 100% tested; whilst cells cultured on Matrigel presented a significant decrease in beat rate and APD80 prolongation and EADs that transitioned to TdPs in just 16% tested. Syncytia cultured on CELLvo had APD80 prolongation, but no incidence of early-afterdepolarizations (EADs) or TdPs. (**C**,**D**) Treatment with 100 µM ranolazine, a safe drug did not change spontaneous beat rate in hiPSC-CMs cultured on Matrigel (n = 6, Δ beat rate = 0 Hz; p = 0.485), CELLvo (n = 6, Δ beat rate = − 0.1 Hz; p = 0.109) or Matrix Plus (n = 6, Δ beat rate = − 0.02 Hz; p = 0.817); Additionally, after ranolazine treatment cardiomyocyte syncytia cultured on Matrix Plus presented APD80 prolongation (n = 6, Δ APD80 = 66.3 ms; p = 0.009) without presence of EADs or TdPs, similar results were obtained with syncytia cultured on CELLvo (n = 6, Δ APD80 = 52.4 ms; p = 0.014) and Matrigel (n = 6, Δ APD80 = 77.9 ms; p < 0.001). (**E**) Microelectrode recording of spontaneous action potentials was done to quantify membrane potentials. Action potential upstroke velocity was greater on cells cultured on Matrix Plus in comparison to Matrigel (p = 0.002). Conduction velocity was significantly higher on Matrigel than Matrix Plus (p = 0.01).

**Figure 3:**
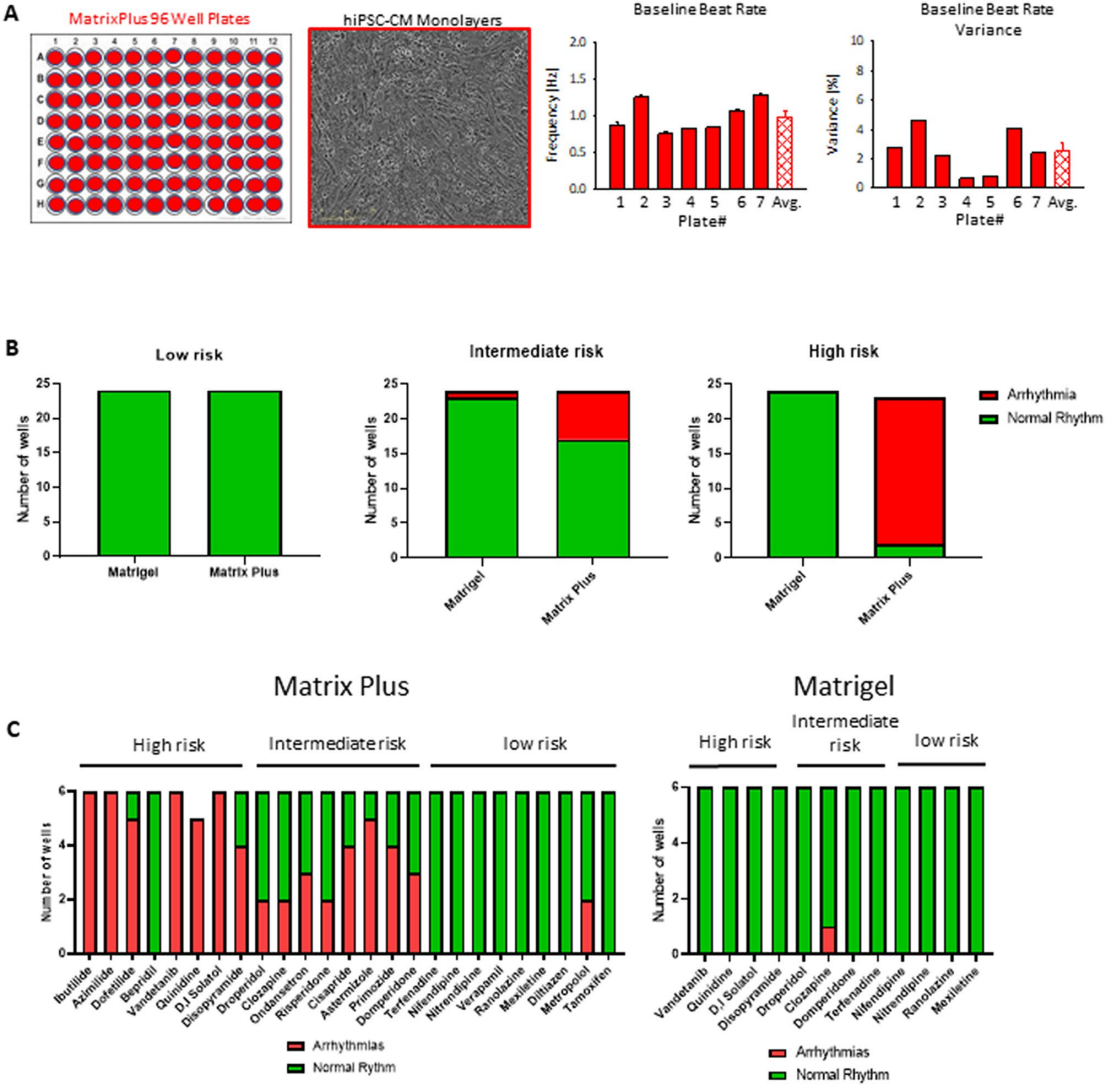
Matrix Plus high-throughput screening assay allows observation of arrhythmias. (**A**) Ninety-six well plates coated with Matrix Plus were submitted to optical mapping for voltage changes and presented a baseline beat rate variation below 6%. (**B**) hiPSC-CMs cultured on Matrigel were treated with drugs classified as high, intermediate and low risk at 10 × ETPC (historical data) were compared to hiPSC-CMs cultured on Matrix Plus and treated with a matched set of drugs at the same concentration. Whilst most of drugs were not able to induce arrhythmias at 10 × ETPC in syncytia cultured on Matrigel, there was a progressive increase in the number of arrhythmias associated with increase in drug risk in the syncytia cultured on Matrix Plus. (**C**) Syncytia culture on Matrix plus were treated with 25 different drugs classifies as high, intermediate and low risk and there was a proportionated increase in the number of arrhythmias corresponding directly to the risk group; nevertheless, increase in arrhythmias was not observed in syncytia cultured on Matrigel.

**Figure 4:**
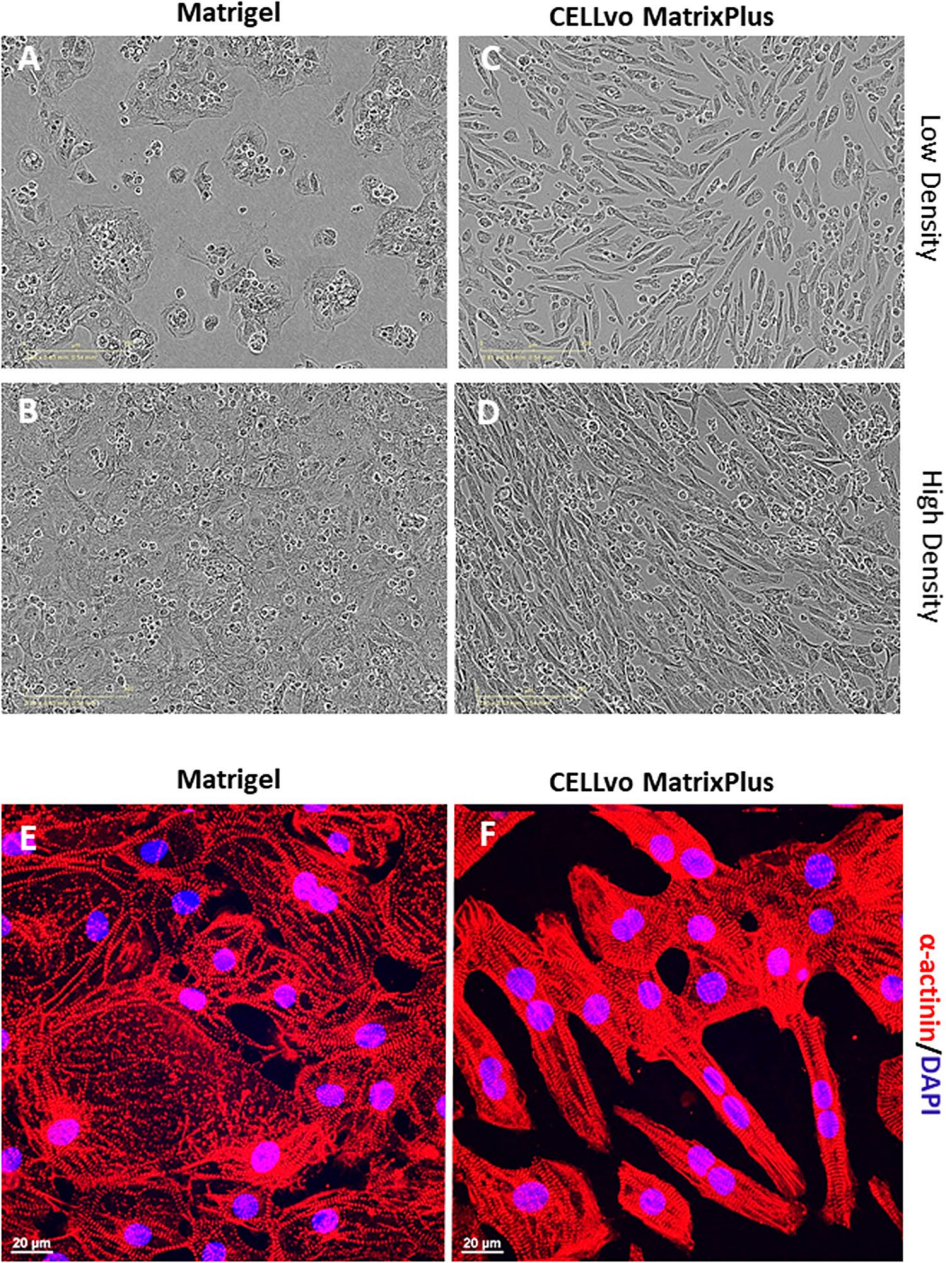
Matrix Plus promotes structural maturation of hiPSC-CMs in 7 days. (**A**,**C**) Bright-field imaging of hiPSC-CM plated in low density shows that Matrix Plus enables attachment comparable to Matrigel, with morphological differences despite using the same batch of cells for each condition. (**B**,**D**) Matrix plus also permits the formation of higher density functional syncytia with preservation of morphology changes that were not observed on Matrigel. (**E**,**F**) Sarcomere staining using α-actinin shows morphological maturation of hiPSC-CMs induced by Matrix Plus.

**Figure 5:**
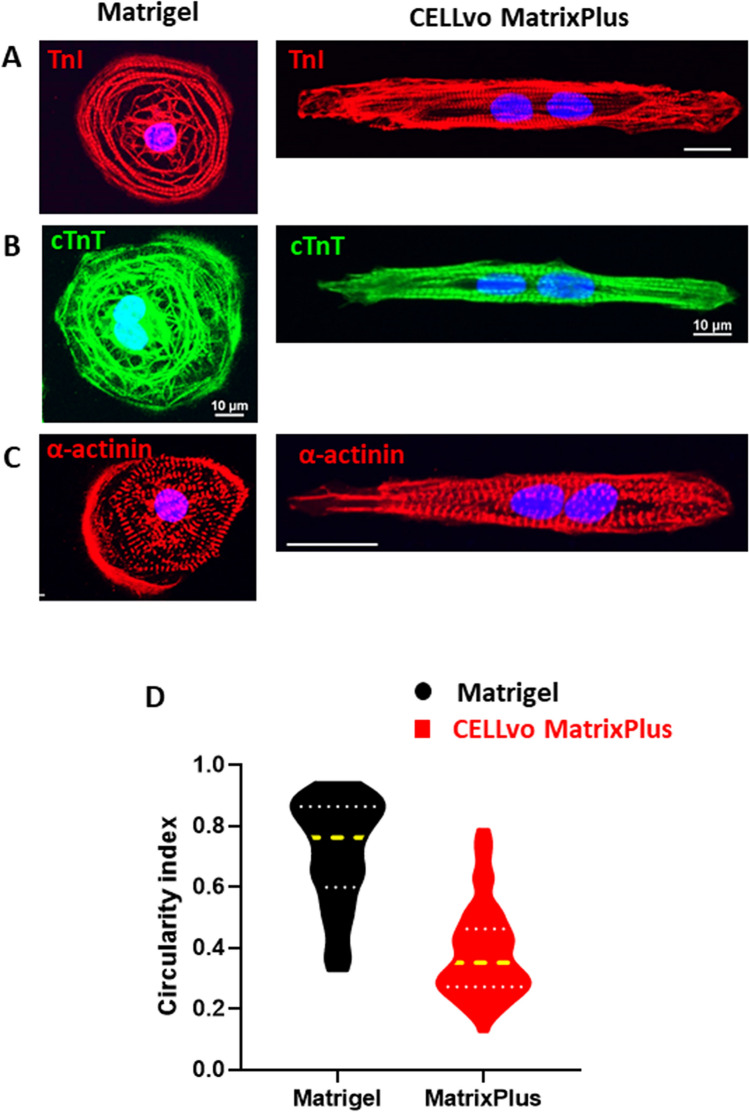
Matrix plus induces morphology changes compatible with adult-like phenotype. (**A**–**C**) TnI, cTnT and α-actinin immunostaining collectively indicate Matrix Plus induces rod-shape morphology with organization of sarcomeres parallel to the long axis of the cardiomyocyte. (**C**) hiPSC-CM circularity index was significantly higher on Matrigel (n = 52; circularity index: 0.71 ± 0.02) than on Matrix Plus (n = 88; circularity index: 0.38 ± 0.01; p < 0.0001). (**D**) Circularity index was calculated using fluorescent images of sarcomere staining. The circularity index for hiPSC-CMs was greater on Matrigel than on Matrix Plus (n = 52, 88 for Matrigel and Matrix Plus respectively, P < 0.0001, unpaired t-test).

**Figure 6:**
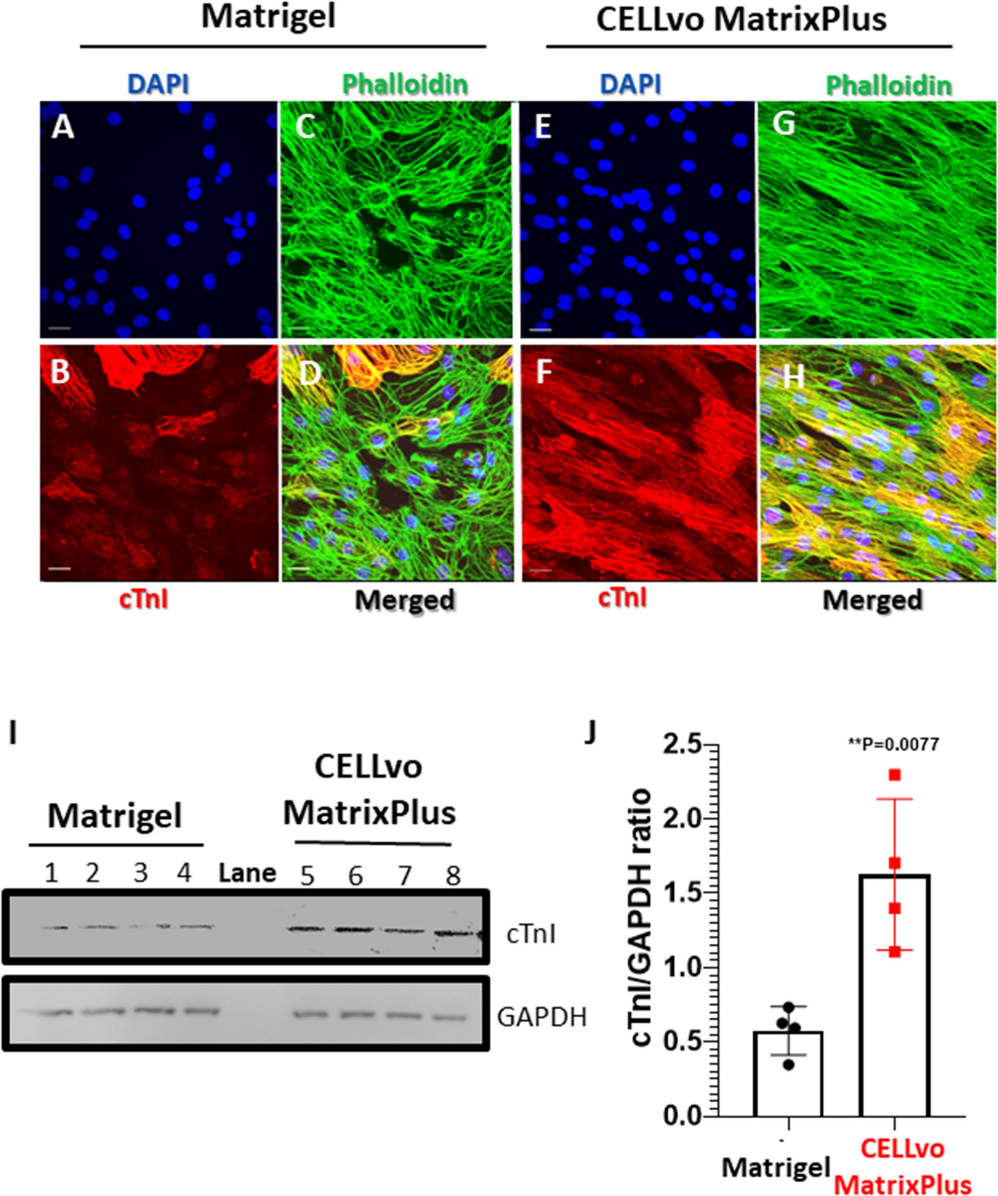
Matrix plus induces higher expression of cTnI, a cardiomyocyte maturation marker. (**A**–**H**) Matrix Plus promoted alignment of actin filaments from cell to cell (phalloidin) and more ubiquitous cTnI expression. (**I**,**J**) Matrix Plus induced ~ 3X greater cTnI protein expression: Matrix Plus (n = 4; cTnI/GAPDH = 1.66 ± 0.25 a.u.); Matrigel (cTnI/GAPDH = 0.58 ± 0.08; p = 0.006). Each blot is a cropped image of the MW for each protein. Full length membranes are in the supplemental material.

**Figure 7:**
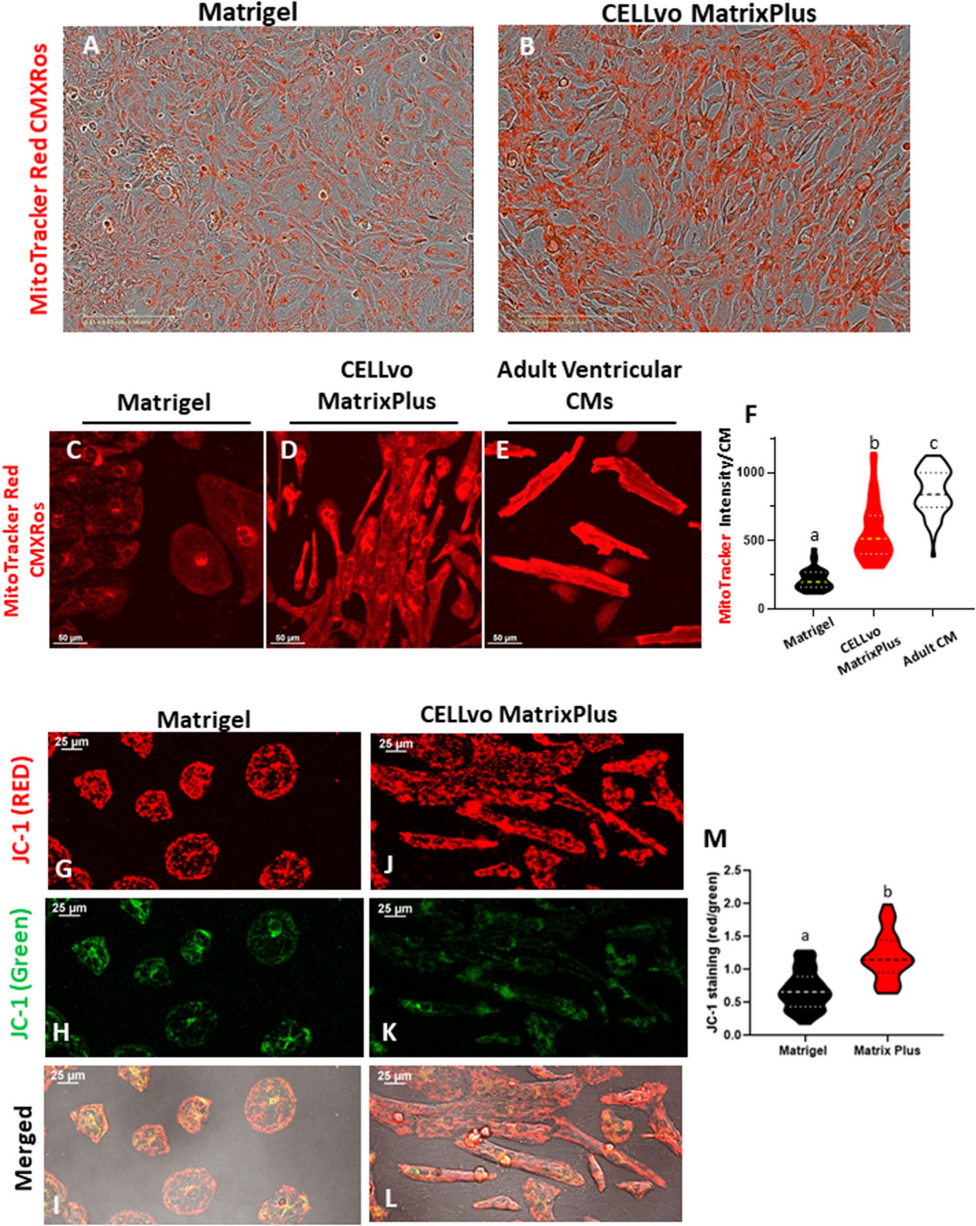
Matrix Plus promotes higher mitochondrial membrane potential. (**A**–**F**) hiPSC-CMs cultured on Matrigel (n = 30, mitrotracker intensity/cell: 221 ± 14.75 a.u.; p < 0.0001) have a lower mitochrondrial content in comparison to cardiomyocytes cultured on Matrix Plus (n = 40, mitotracker intensity/cell: 569 ± 34.9a.u.) and murine adult ventricular cardiomyocytes (n = 56, mitotracker intensity/cell: 859 ± 22.3a.u.).(**G**–**M**) Mitochondria functional information obtained with JC-1 also indicates that cardiomyocytes cultured on Matrix Plus have higher mitochondrial membrane potential than cardiomyocytes cultured on Matrigel (p < 0.0001).

Figures 2, 3, 4, 5, 6 and 7 demonstrate that Matrix Plus supports functional and structural maturation of hiPSC-CMs in culture to a much greater extent than other ECMs tested. In these experiments cryopreserved hiPSC-CMs (Cellular Dynamics International, iCell^2^) were plated as electrically and mechanically connected monolayers on one of three ECMs: 1. Matrix Plus, 2. CELLvo Matrix, or 3. Matrigel. hiPSC-CM monolayers were given 7 days to mature and functional analysis was done to determine electrophysiology characteristics and drug responsiveness. Only hiPSC-CM monolayers plated on Matrix Plus showed Torsades de Pointes (TdP) like arrhythmias characterized by stable rotors in the presence of hERG channel blockade with E-4031 in 100% of monolayers tested (Fig. 2A, B; supplemental movie 1). Matrigel (a commonly used commercially available ECM isolated from murine source) and Matrix Plus have presented the greatest potential to detect pro-arrhythmia effects of E-403, in summary, cells culture on Matrigel and Matrix Plus presented higher incidence of TdP at lower amplitudes of APD80% prolongation. Additionally, we have observed that cardiomyocytes cultured on matrigel and CELLvo matrices did not present drug-induced alteration of conduction velocity; however, cells cultured on Matrix Plus presented significant TdP-associated slowing of conduction velocity (Fig. 2B), recapitulating slowing of conduction velocity that follows arrhythmias in the heart and contributes to the permanence of an arrhythmic state^30–33^. Ranolazine treatment did not significantly alter beat rate or conduction velocity, and despite of ranolazine-mediated APD80% prolongation, we did not observe appearance of arrhythmias of TdP in any of the matrices tested (Fig. 2C,D), indicating that this system did not yield false positive results for ranolazine. Based on the observation on E-4031 and ranolazine treatment, we opted to restrict low through-put patch clamp experiments to Matrigel and Matrix Plus. On Matrix Plus, hiPSC-CM action potential profiles mimicked adult cardiomyocyte values with hyperpolarized diastolic potentials, rapid action potential upstroke velocity and action potential conduction velocity more consistent with adult CM phenotype (Fig. 2E, table).

### hiPSC-CM 96 well plate HTS electrophysiology validation using VSD

Next, Matrix Plus was produced on high throughput screening plates (96 well plates) for HTS electrophysiological effects of medications with known risk to cause TdP in patients (Fig. 3A–C; supplemental movie 2). Seven Matrix Plus plates were used and the coefficient of variance of beat rate was determined to be well below 10% from plate to plate, thus showing the suitability of Matrix Plus for HTS assays. Proarrhythmia/cardiotoxicity screening was done using the recently described CiPA validation compounds published by Blinova et al.^5^ with 24 compounds of known clinical risk to cause TdP (Fig. 3B). hiPSC-CM syncytia cultured on Matrix Plus presented a significantly higher percentage of with arrhythmias when treated with high risk drugs (mean ± SEM: 81.25% ± 12.37% arrhythmias); in fact, 5 out of 8 high risk drugs presented arrhythmias in all treated wells, with the exception of dofetilide that presented 83% of the wells with arrhythmias, disopyramide that presented 66% of the wells with arrhythmias and bepridil that did not yield arrhythmic responses. Intermediate risk drugs presented a lower number of wells with arrhythmias (46.22% ± 8.25% arrhythmias); however, all intermediate drugs were able to induce arrhythmias. Nine out of 8 low risk drugs presented normal rhythm when treated at 10 × ETPC (4.1% ± 4.1% arrhythmias). Matrix Plus hiPSC-CM monolayers responded as expected to 24 compounds with known risk to cause TdP arrhythmias based on clinical data (Fig. 3B, supplemental Tables 1–3). Syncytia culture on matrigel did not present arrhythmias when treated with a subset of the CiPA validation compounds (4 drugs in each risk group) at 10 × ETCP, with the exception of clozapine that had 1 well presenting arrhythmia (Fig. 3B). Finally, Matrix Plus and Matrigel results were matched by drugs and sorted into risk groups for comparison. None of syncytia cultured on matrigel and Matrix Plus presented arrhythmias after treatment with10x ETCP of low risk drugs. However, syncytia cultured on Matrix Plus presented a significant higher proportion of wells presenting arrhythmias than those culture on matrigel after exposure to 10 × ETPC of intermediate risk drugs (matrigel – 4.7%; Matrix Plus – 29.17%; p = 0.0479). The highest discrepancy in arrhythmogenic response between syncytia cultured in the two different matrices was observed with high risk drugs. None of the syncytia cultured on matrigel presented arrhythmias (0%), although most of the syncytia cultured on Matrix Plus presented arrhythmias (91.3%; p < 0.0001).

### Matrix plus ECM promotes hiPSC-CM structural maturation

Matrix Plus supports structural hiPSC-CM maturation (Figs. 4, 5, 6, 7). A hallmark of adult cardiomyocytes is their unique rod shape morphology with alignment from cell to cell. Figures 4 and 5 show extensive analysis of hiPSC-CM morphology using phase contrast and confocal imaging of immunoassayed CMs. Circularity index of single cells was significantly lower for hiPSC-CMs cultured on Matrix Plus compared to Matrigel ECM (Fig. 5D, violin plots). Supplemental Fig. 2 shows that the characteristic light dark repeating pattern of muscle sarcomeres is visible on phase contrast imaging in rod shaped cells cultured on Matrix Plus. Further, large fields of view images show rod shaped morphology of hiPSC-CMs cultured on Matrix Plus, while the same batch of cells cultured on Matrigel retained a circular shape with disorganized sarcomeres (TnI). Images of monolayers on Matrix Plus indicate also that hiPSC-CMs adopt a natural alignment of cytoskeleton from cell to cell as indicated by phalloidin staining of cellular actin filaments (green, Fig. 6G). Importantly, using hypertrophic cardiomyopathy disease specific cells characterized by sarcomere and cellular disarray, the disease phenotype was still apparent when cultured on Matrix Plus (supplemental figure 3). Matrix Plus induced greater expression of the mature myofilament marker cTnI (red, Fig. 6F and Western Blot Fig. 6I–JF). Mature myofilament development is also apparent by the appearance of the light–dark repeating pattern of developed sarcomeres observed by phase contrast imaging (supplemental figure 4) and upon immunostaining for cardiac troponin T (cTnT, supplemental figure 5).

Figure 7 shows that Matrix Plus also matures hiPSC-CM mitochondrial distribution and function to levels more like adult cardiomyocytes. We further investigated differences in mitochondrial membrane potential of cardiomyocytes cultured on Matrigel and Matrix Plus and observed that cells cultured on Matrix Plus presented a higher mitochondrial membrane potential than those cultured on Matrigel (Fig. 7F, additional images in supplemental figure 6). This observation was confirmed with staining of hiPSC-CMs cultured in both matrices with JC-1 mitochondrial specific fluorescent marker. JC-1 accumulates in the mitochondria and presents dual fluorescence (green at low mitochondrial membrane potential and red at high mitochondrial potential). hiPSC-CM cultures on Matrix Plus exhibited a significantly higher mitochondrial potential in comparison to those cultured on matrigel (red/green ratio of Matrix Plus: 1.194 ± 0.362; red/green ratio of matrigel:0.688 ± 0.296, p < 0.0001).

## Discussion

Our work presents a solution to a current problem facing the advancement of in vitro cardiotoxicity screening using human cardiomyocytes (hiPSC-CMs), namely the immaturity of the cells. Here we tested the hypothesis that human cell derived ECM can promote hiPSC-CM maturation in 2D monolayer formats. To this end we produced and validated a novel human perinatal stem cell derived ECM that we call Matrix Plus. Results indicate that Matrix Plus supports rapid maturation (7 days) of hiPSC-CM functional and structural phenotypes. Furthermore, we validated Matrix Plus ECM use in a VSD proarrhythmia screen using 96 well plates. Matrix Plus ECM is reproducible from batch to batch (Supplemental figure 1), has a long shelf life and is easy to rehydrate and use for cell culture. Matrix Plus provides a simple and efficient solution to the well-recognized problem of stem cell derived cardiomyocyte maturation^34^.

ECM mediated maturation can occur through mechanical cues and\or biochemical components of the matrix. Matrix Plus ECM mechanical properties are distinct from other human cell-derived ECM coatings. Figure 1 outlines the differences of ECM properties derived from distinct human cell sources. Matrix Plus ECM is derived from perinatal stem cells while CELLvo Matrix ECM is derived from bone marrow cells. The ECM cell source (bone vs. stem cell) has impact on the hardness and stiffness of the ECM used for cell culture. Indeed, atomic force microscopy analysis revealed higher adhesive forces, but lower stiffness of Matrix Plus relative to CELLvo Matrix (Fig. 1). Surface energy related adhesive forces can impact early cell attachment and spreading, hydration and uptake of soluble factors^35^, while matrix stiffness can affect a host of cell behaviors and plays a critical role in cardiomyocyte cytoskeletal organization, contractility and maturation^17,36^. These mechanical cues may underlie the maturation observed using Matrix Plus, but not CELLvo Matrix.

Biochemical analysis of each human cell derived ECM revealed 69% similarity in biomolecular composition between the substrates. A major difference of interest is the different abundance of perlecan between the two ECMs. Perlecan is a major component of basement membranes and critical in the development of a variety of structures from every germ layer^37,38^. Mutations in perlecan are lethal to the developing embryo at ED10-12 due to blood hemorrhaging into the pericardial cavity, while less severe mutations cause death perinatally. It is possible that a mutation causing death perinatally, may result from a disruption of cardiac morphogenesis, i.e. dysfunction of cardiomyocytes maturation and organization^39^. Thus, the relative abundance of perlecan in Matrix Plus may be a molecular mechanism critical to the observed maturation phenomenon. Overall, our analysis highlights several interesting differences between CELLVo Matrix and Matrix Plus that may be responsible for the unique cardiomyocyte maturation phenomenon observed on Matrix Plus. This observation illustrates a broader point about cell culture substrates-interactions between cells and their substrates are nuanced and critically important for in vitro screening assays. Based on these data, it is tempting to assume that we can easily identify appropriate physical properties and critical biochemical attributes and recreate this phenomenon using a synthetic substrate and recombinant proteins. While it may be possible to identify critical components by knocking down expression of certain matrix proteins, blocking binding, or otherwise inhibiting known pathways, it is overly simplistic to assume that a mechanism of action may be readily determined based on these complex cell matrix interactions. It is more likely that the observed phenomenon is the result of an “entourage effect” where the combined result is greater than the sum of its parts. As such, it is possible that This concept at the pitfalls of attempting to simplify a cell niche are illustrated quite clearly in Herron et al.^25^, where it was demonstrated that Matrigel promoted greater cardiac maturation than any of its constituent proteins on their own, or Ragelle et al., where analysis of three distinct cell-derived matrices did not identify likely mechanisms for differential cell responses to those matrices.

Maturation of cardiomyocytes can be obtained in 2D or 3D culture systems^12,25,40^. 3D cell culture systems have inherent limitations and production of engineered heart tissues (EHT) vary widely due to discrepancies in 3D culture systems used by different groups. Additionally, EHT assays are inherently low-throughput and this limitation prevents the adoption of 3D assays in the drug discovery pipeline. In this context, maturation of 2D monolayers for high-throughput drug screening of new therapeutic components is an urgent topic. Heretofore advanced 2D maturation of hiPSC-CM monolayer electrophysiological function utilized Matrigel coated PDMS coverslips^17^. Further, advanced maturation of single hiPSC-CMs in 2D cell culture utilized a “Matrigel Mattress” approach^13,36^. Matrigel is a mouse derived ECM comprised of many matrix proteins and signaling molecules and promoted hiPSC-CM maturation to a greater extent than using purified protein ECM coatings like collagen, fibronectin or laminin^17^. This suggests that an “entourage effect” of multiple ECM signaling inputs is required to promote maturation of hiPSC-CMs. Matrix Plus, made up of many ECM components (Fig. 1) represents a human cell derived matrix that retains the precise structural and chemical composition designed by cells. hiPSC-CMs respond to the Matrix Plus ECM environment by rapid maturation of CM structure and function (Figs. 2, 3, 4, 5, 6, 7). hiPSC-CM phenotypes were compared directly to cells cultured on Matrigel ECM to compare human cell derived matrix to mouse derived matrix. hiPSC-CM maturation was more apparent on human cell derived Matrix Plus.

In electrophysiology assays, hERG channel specific blockade (E-4031) caused drug-initiated rotors or TdP like arrhythmias in 100% of hiPSC-CM monolayers tested on Matrix Plus (supplemental movie 1). This result is of utmost importance and represents an advance in assays to screening new drugs for TdP risk and an additional readout beyond the surrogate marker of action potential duration. The presence of stable rotors initiated by E-4031 in monolayers cultured on Matrix-plus was followed by slowing of conduction velocity recapitulating the slowing in conduction velocity observed in fibrilatory conduction. The ability to detect drug-initiated reentrant arrhythmias in 2D cardiomyocytes preparations differs fundamentally from pacing-initiated arrhythmias (Laksman 2017, Kadota 2013) because drug-initiated arrhythmias are caused by drug-induced alterations in ion channel gating properties, and rapid pacing-initiated arrhythmias are caused by temporal overlap of electrical stimulus and cardiomyocyte refractory period. Rapid pacing-initiated arrhythmias have an important role in investigating anti-arrhythmic properties of drugs but have limited value for determining the QT prolongation arrhythmogenic potential of a new compound.

Importantly the proarrhythmia assay here using Matrix Plus did not detect any arrhythmias in ranolazine treated hiPSC-CM monolayers (Fig. 3). In recent FDA validation reports for hiPSC-CM assays ranolazine was an outlier drug that incorrectly produced arrhythmias in hiPSC-CMs^5^. This offers an advantage of using Matrix Plus ECM for EP assays to correctly assign proarrhythmia risk for compounds. Furthermore, most assays to determine arrhythmogenic risk utilize surrogate measures for arrhythmias such as prolongation of action potential duration, or rely on complex models that include several electrophysiology parameters (Blinoa 2018 monteiro da rocha 20 jicsa2018). Blinova et al. suggests that production of adult-like hiPSC-CMs may increase the predictive power of assays to detect arrhythmogenesis and that new parameters might be included in risk models. Matrix plus is able to deliver hiPSC-CMs that have a phenotype closer to adult like cells and provides direct detection of arrhythmias in a risk stratified fashion. Nevertheless, calculation of a complete model to predict arrhythmogenesis using Matrix Plus and the incorporation of risk stratified direct detection of arrhythmias may require a large scale multi-center study that is out of the scope of the current manuscript. Matrix Plus promoted rapid structural maturation of hiPSC-CMs as presented in Fig. 3. Multiple batches of Matrix Plus had the same effect to promote the rod-shaped morphology of hiPSC-CMs in just 7 days of cell culture maintenance after thaw. hiPSC-CMs cytoskeletal structure alignment was apparent from cell to cell in monolayers grown on Matrix Plus relative to Matrigel ECM. This feature of control hiPSC-CMs to align on Matrix Plus is important for modeling diseases such as hypertrophic cardiomyopathy (HCM). Supplemental figure 1 shows that HCM patient disease hiPSC-CMs adhere to Matrix Plus, but cytoskeletal and sarcomeres are disorganized-which recapitulates the clinical phenotype of HCM.

The structural maturation of hiPSC-CMs on Matrix Plus may have implications for cardiotoxicity screening assays. For example, use of high sensitivity cardiac troponin I (cTnI) biomarker for detection of cardiac damage will only be useful if hiPSC-CMs are expressing significant amounts of cTnI. cTnI expression is induced in hiPSC-CMs grown on Matrix Plus. Mitochondrial maturation induced in hiPSC-CMs grown on Matrix Plus will also have implications for detecting cardiotoxicity of compounds that act through mitochondrial pathways to exert toxic effects. Immature hiPSC-CMs with diminished distribution and functional mitochondria (Fig. 3) may not reveal toxic effects of compounds that act on mitochondrial functions and pathways. Matrix Plus effects to mature hiPSC-CM phenotypes will have impact on the outcome of long-term chronic studies of drugs to induce cardiotoxicity.

## Supplementary information

Supplementary Information.

Supplementary Video 1.

Supplementary Video 2.
