# Supplementary material for: Human perinatal stem cell derived extracellular matrix enables rapid maturation of hiPSC-CM structural and functional phenotypes
Source: Sci Rep. 2020 Nov 4;10:19071. doi: 10.1038/s41598-020-76052-y (PMC7643060; doi:10.1038/s41598-020-76052-y)
Supplement: Supplementary file 1 — Supplementary Information. [file 41598_2020_76052_MOESM1_ESM.docx]

*Human perinatal stem cell derived extracellular matrix enables rapid maturation of hiPSC-CM structural and functional phenotypes*

Travis Block^1^, Jeffery Creech^2,3^, Andre Monteiro da Rocha^2,3^, Milos Marinkovic^4^, Daniela Ponce- Balbuena^2^, EN Jiménez-Vazquez^2^, Sy Griffey^1^, Todd J. Herron^2,3,¥^

^1^StemBioSys, Inc, 3463 Magic Drive, Suite 110, San Antonio, TX 78229

^2^Frankel Cardiovascular Regeneration Core Laboratory, 2800 Plymouth Road, University of Michigan, Ann Arbor, 48109

^3^Cartox, Inc., 1600 Plymouth Road, B520 2^nd^ floor, Ann Arbor, MI 48109

^4^University of Texas Health at San Antonio, Department of Comprehensive Dentistry, San Antonio, TX USA

¥Corresponding Author: Todd J. Herron, University of Michigan North Campus Research Complex, 2800 Plymouth Road, B26 223N, Ann Arbor, MI 48109 [toddherr@umich.edu](mailto:toddherr@umich.edu)

**Supplemental Material**

Supplemental tables 1-3 outline each drug tested, the CiPA risk classification, the concentrations used and the clinical recorded Cmax for each drug to provide clinical reference for drug concentrations. hiPSC-CM monolayers were then submitted to optical mapping for detection of membrane voltage changes recorded with the CCD camera via selection of appropriate light wavelength (filter 515 nm, green light, Chroma). Movies were analyzed with custom made analysis software (Scroll) for determination of action potential duration and conduction velocity where appropriate.


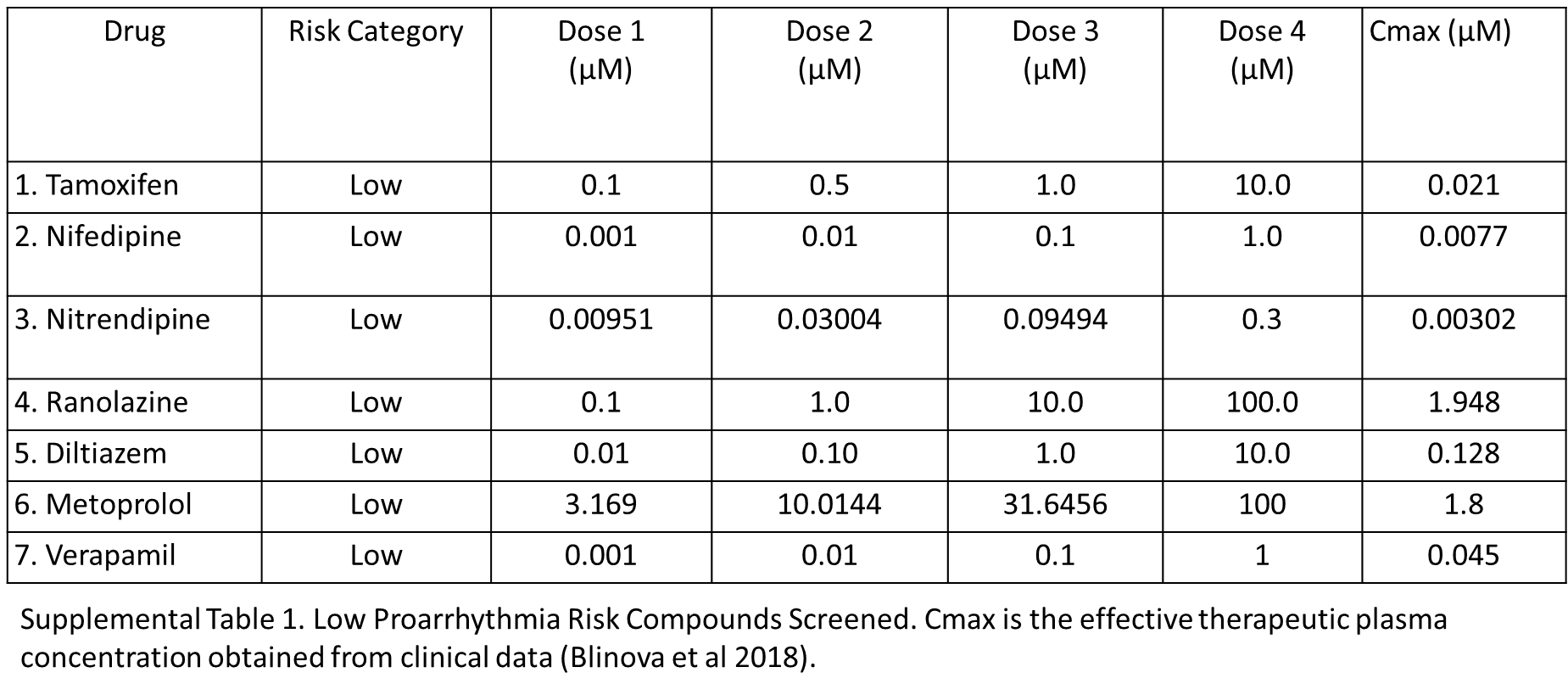


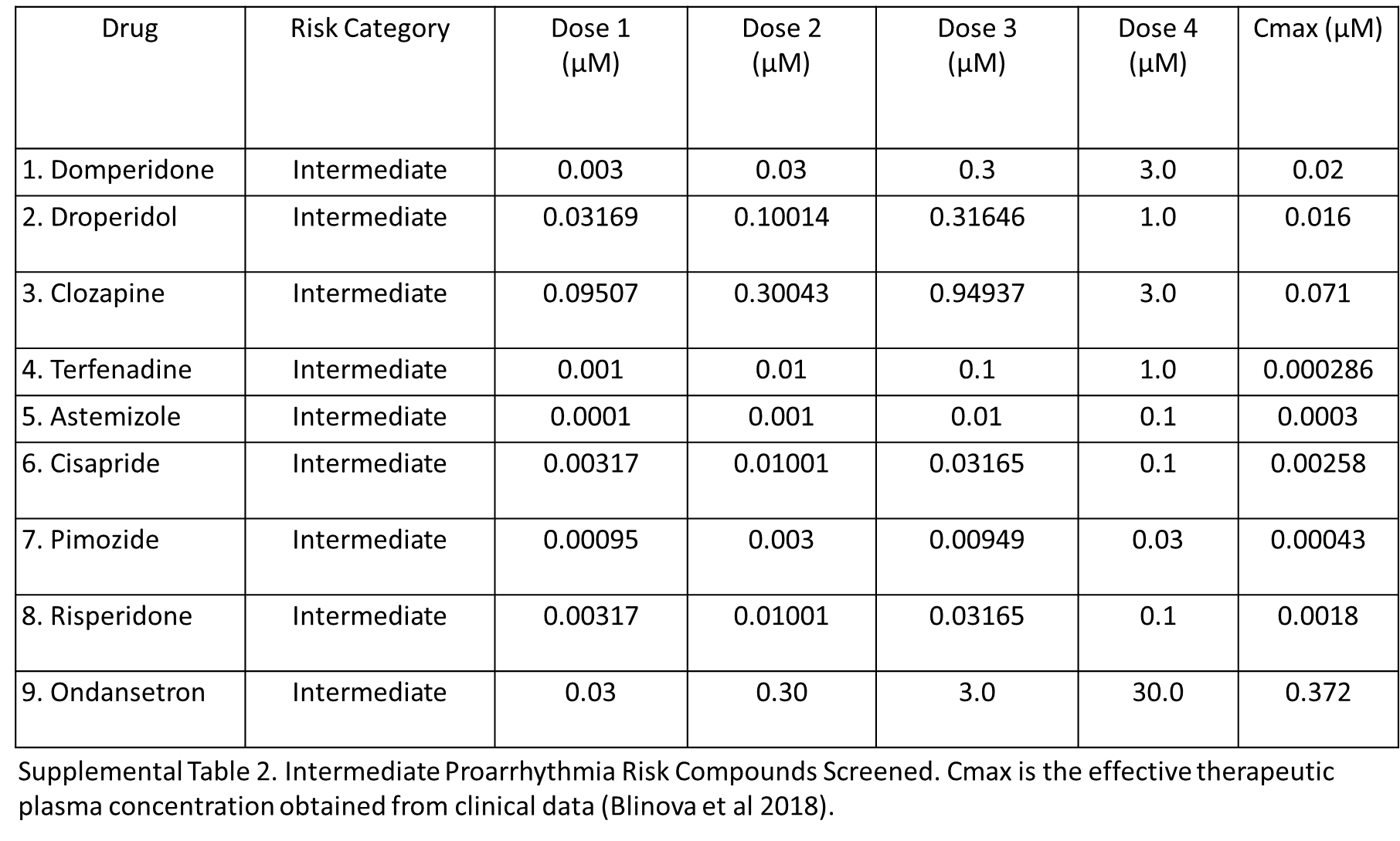


Supplemental Table 2. Intermediate Proarrhythmia Risk Compounds Screened. Cmax is the effective therapeutic plasma concentration obtained from clinical data (Blinova et al 2018).


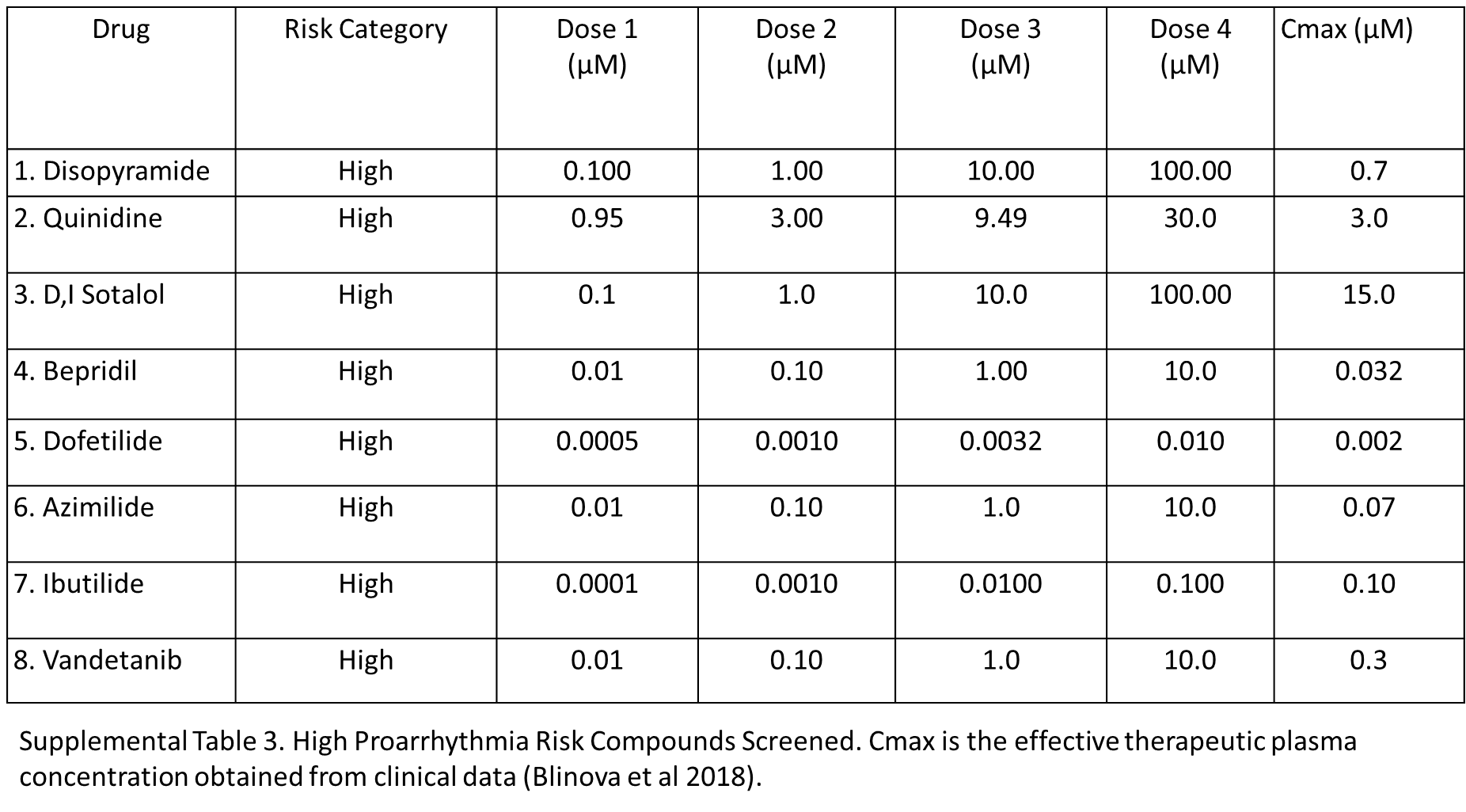


**Antibodies used for hiPSC-CM Structural Analysis**

| **Antibody/Fluorophore** | **Vendor/Supplier** | **Cataloge number** |
| --- | --- | --- |
|  |  |  |
| cTnI | Abcam | Ab10231 |
| α-actinin | Sigma | A7811-2mL |
| pan-TnI | Milipore | MAB1691 |
| cTnT | Abcam | Ab10214 |
| phalloidin-488 | InVitrogen | A12379 |
| DAPI | Sigma | D1306 |
| GAPDH | Sigma | G9545-250μL |
| AlexaFluor 488 conjugated ab | ThermoFisher | A32723; A32731 |
| AlexaFluor 594 conjugated ab | ThermoFisher | A32740; A32742 |
| MitoTracker Red CMXRos | Fisher scientific | M7512 |

Supplemental Table 4. Antibodies and fluorescent labels used to characterize hiPSC-CM structure.

**Supplemental Figure 1.** Principal component analysis of composition of Matrix Plus (MP) & CELLvo Matrix. 3 unique donors were used to produce CELLvo Matrix or Matrix Plus. Total spectral counts from mass spectrometry were analyzed using principal component analysis using singular value decomposition with imputation to calculate principal components. X and Y axis show principal component 1 and principal component 2 that explain 75% and 11.4% of the total variance, respectively. N = 6 data points.


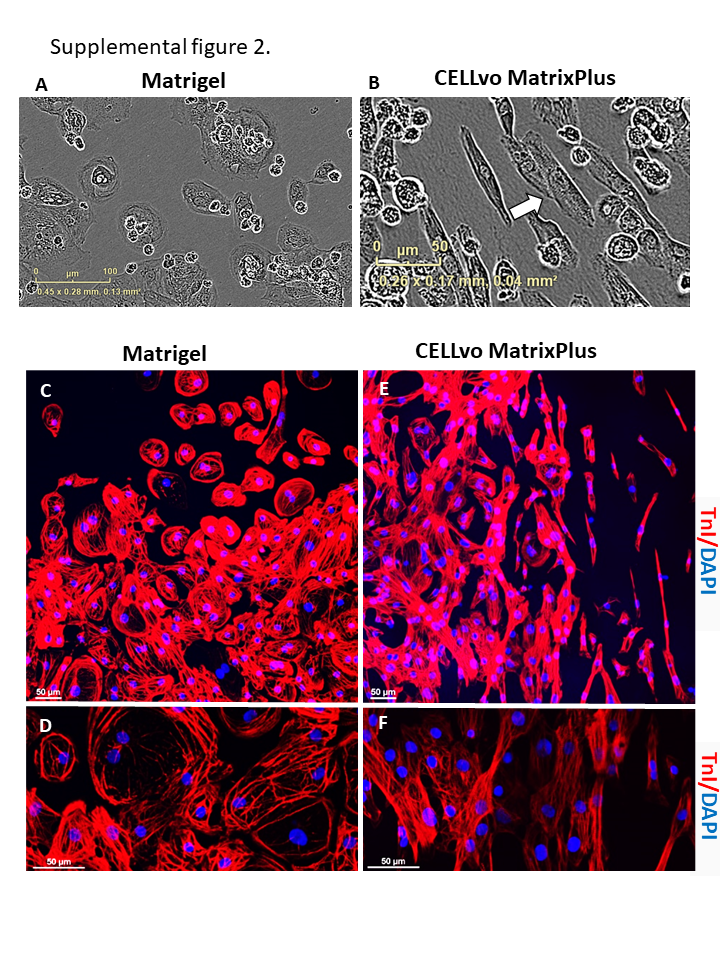


***Supplemental Figure 2.*** A, Phase Contrast images of hiPSC-CMs cultured on Matrigel, and B, on Matrix Plus. In B, the arrow points to the observation of sarcomeres upon simple phase contrast imaging. C-D, total troponin I staining (TnI) for hiPSC-CMs cultured on Matrigel. E-F, total TnI staining for hiPSC-CMs cultured on Matrix Plus.


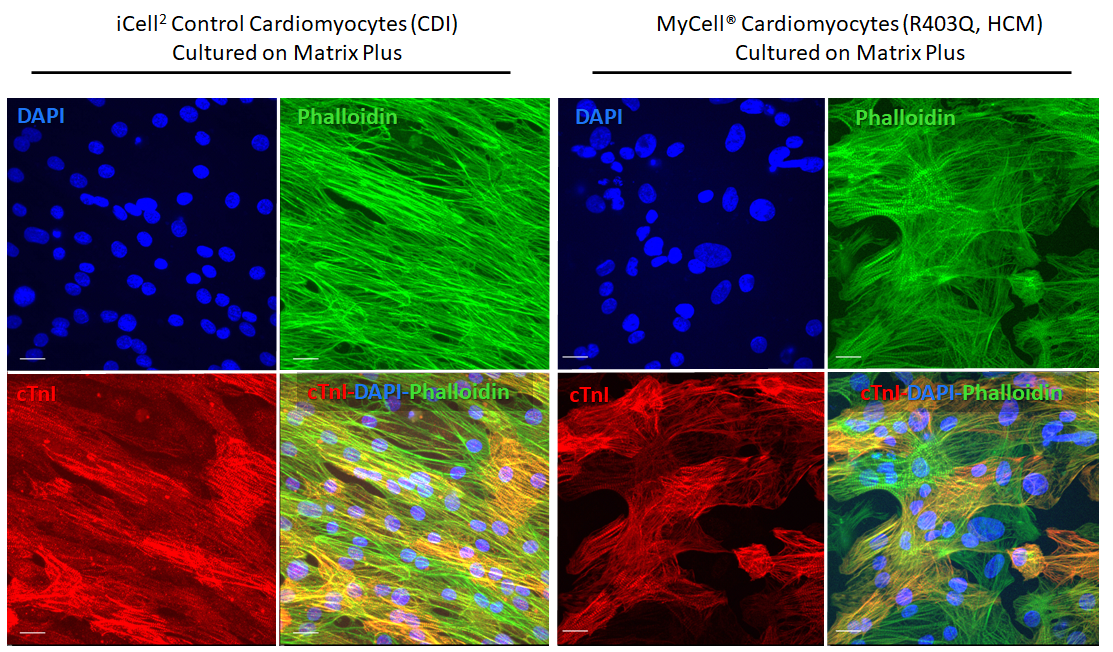


***Supplemental figure 3.*** Control hiPSC-CMs (left, same image as in main text figure 6) cultured on Matrix Plus align well from cell to cell, indicated by phalloidin staining (green). On the other hand, Hypertrophic cardiomyopathy (right, HCM, R403Q) disease specific cells do not align on Matrix Plus. cTnI staining also shows the differences of cellular alignment. The misalignment of sarcomeres and cellular disarray are hallmark for HCM. This is important to show that disease phenotypes can be recapitulated on Matrix Plus.

***
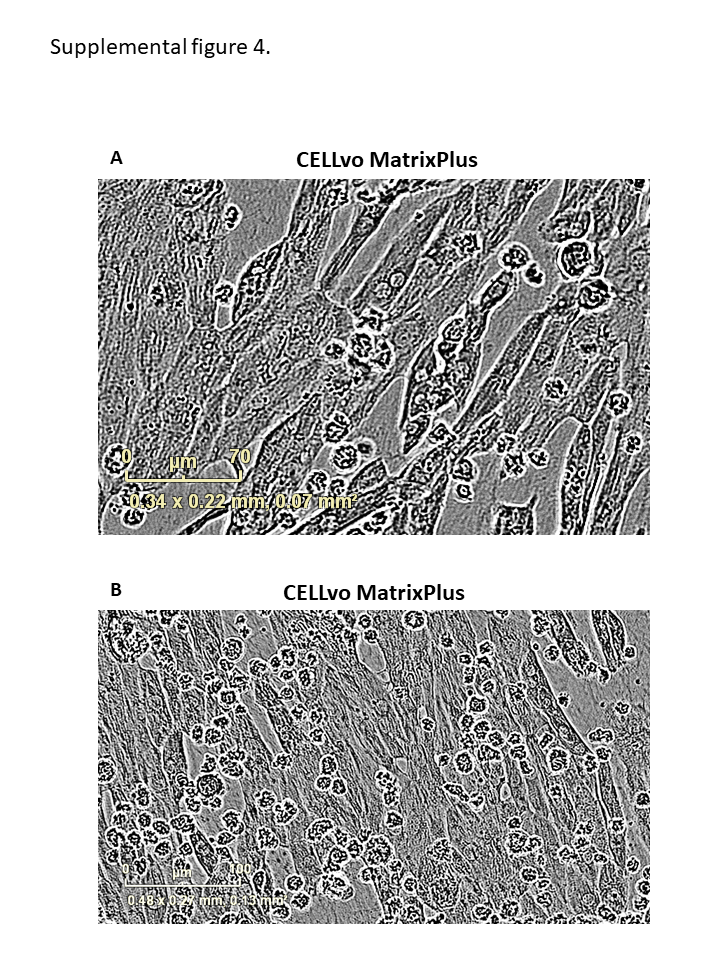
***

**Supplemental Figure 4.** Phase Contrast images of hiPSC-CMs cultured on Matrix Plus (A and B). These images show the cell morphology and also indicate the observation of sarcomeres under phase contrast imaging. 20X, incucyte imager.

***
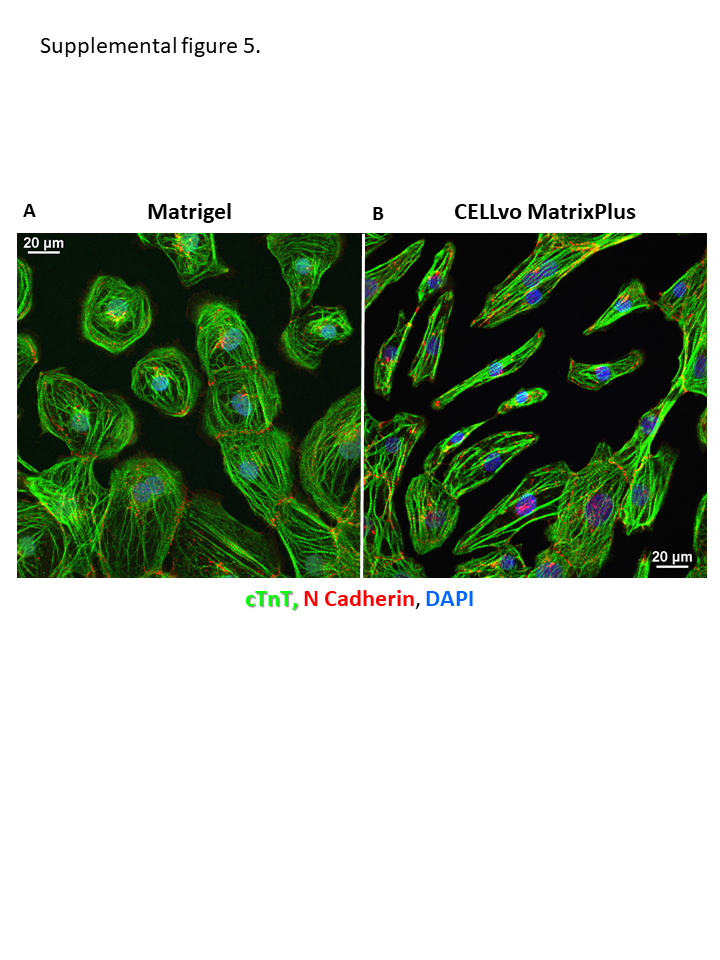
***

***Supplemental figure 5.*** Confocal images of hiPSC-CMs stained for cTnT, N Cadherin and DAPI. Panel A shows images of cells cultured on matrigel. Panel B shows images of cells cultured on Matrix Plus.


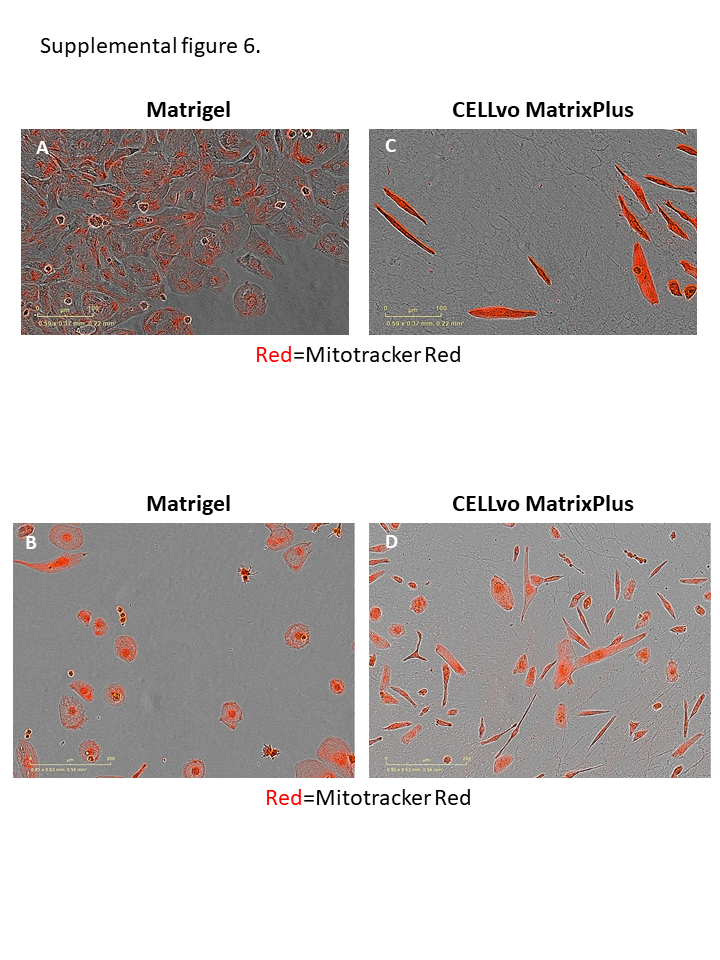


***Supplemental figure 6.*** hiPSC-CMs loaded with Mitotracker Red. A-B, cells cultured on Matrigel. C-D, cells cultured on Matrix Plus.

***
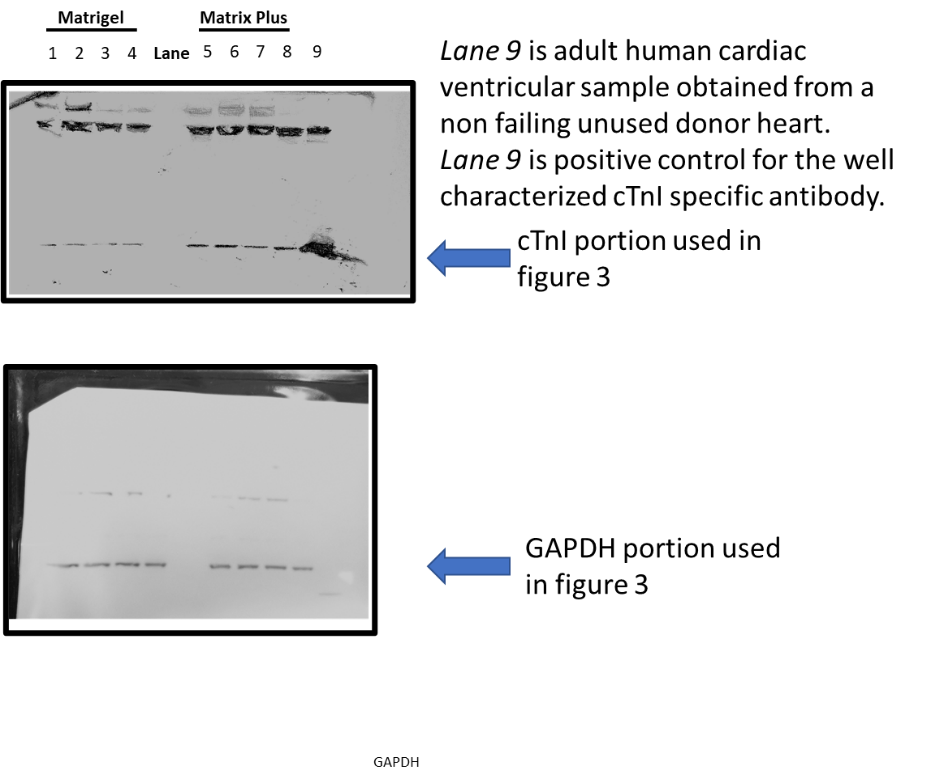
***

6

6

***Supplemental figure 7.*** Full length membrane images for Western Blots in Figure 6I. These experiments used a well established cTnI specific antibody. Nevertheless, we used a protein sample isolated from an adult human heart from a non-failing donor patient as a positive control (lane 9). The heart was not used for transplantation-obtained from Gift of Life Michigan. GAPDH immunoblotting was subsequently performed to determine relative protein load for each well.

***Statistics and Data Analysis***

Statistical analysis was performed with GraphpadPrism and Student’s T test or ANOVA followed by Tukey’s test for means as a post-hoc test was performed as appropriate. Data was expressed as mean±SEM and significance was achieved at p<0.05.
